# Resveratrols in Grape Berry Skins and Leaves in *Vitis* Germplasm

**DOI:** 10.1371/journal.pone.0061642

**Published:** 2013-04-24

**Authors:** Lijun Wang, Man Xu, Chunyan Liu, Junfang Wang, Huifen Xi, Benhong Wu, Wayne Loescher, Wei Duan, Peige Fan, Shaohua Li

**Affiliations:** 1 Key Laboratory of Plant Resources and Beijing Key Laboratory of Grape Science and Enology, Institute of Botany, the Chinese Academy of Sciences, Beijing, China; 2 University of Chinese Academy of Sciences, Beijing, China; 3 College of Agriculture and Natural Resources, Michigan State University, East Lansing, Michigan, United State of America; 4 Key Laboratory of Plant Germplasm Enhancement and Specialty Agriculture, Wuhan Botanical Garden, the Chinese Academy of Sciences, Wuhan, China; TGen, United States of America

## Abstract

**Background:**

Resveratrol is an important stilbene that benefits human health. However, it is only distributed in a few species including grape and is very expensive. At present, grape has been an important source resveratrol. However, the details are scarce on resveratrol distribution in different *Vitis* species or cultivars.

**Methodology/Principal Finding:**

The composition and content of resveratrols were investigated by HPLC for assessing genotypic variation in berry skins and leaves of 75 grape cultivars, belonging to 3 species and 7 interspecific hybrids. *Trans*-resveratrol, *cis*-piceid and *trans*-piceid were detected in berry skins and leaves, but *cis*-resveratrol was not. Resveratrol content largely varied with genetic background as well as usage. In most cultivars, total resveratrol including the above three compounds was higher in berry skins than leaves. In berry skins of most cultivars and leaves of almost all cultivars, *cis*-piceid was the most abundant resveratrol; *trans*-resveratrol and *trans*-piceid were minor components. Some specific cultivars were found with extremely high levels of *trans*-resveratrol, *cis*- piceid, *trans*-piceid or total resveratrols in berry skins or leaves. In skins and leaves, rootstock cultivars had a higher content of total resveratrols, and the cultivated European type cultivars and their hybrids with *V. labrusca* had relatively low totals. There were no significant correlations of the amounts of total resveratrols or any individual resveratrol between berry skins and leaves. All 75 cultivars can be divided into four groups based on the composition of resveratrols and their concentration by principal component analysis.

**Conclusion:**

Resveratrol content of grape berries and leaves varied largely with their genetic background and usage. Rootstock cultivars had a higher content of total resveratrols than the other germplasm. Total resveratrols were lower in leaves than berry skins in most cultivars. *Cis*-piceid was the most abundant resveratrol in most cultivars, and *trans*-res and *trans*-pd were minor components.

## Introduction

The polyphenol *trans*-resveratrol (*trans*-3,5,4′-trihydroxystilbene), a member of the stilbene family, was first isolated from white hellebore (*Veratrum grandiflorum O*.) [1]. It has become the focus of a number of studies in medicine and plant physiology, and has also emerged as a molecule that may affect human health. In leaves and berries of grape (*Vitis*), *trans*-resveratrol is a phytoalexin that is produced in response to stresses, such as wounding or pathogen attack [2]. Due to its daily consumption in the form of red wine [3]–[4], *trans*-resveratrol has been associated with the “French paradox” for having beneficial effects [5], particularly protection against coronary diseases [6]. In addition, *trans*-resveratrol may have anticancer properties, as suggested by its ability to suppress proliferation of a wide variety of tumor cells, including lymphoid and myeloid cancers; multiple myeloma; cancers of the breast, prostate, stomach, colon, pancreas, and thyroid; melanoma; head and neck squamous cell carcinoma; ovarian carcinoma and cervical carcinoma [7]–[8]. Nowadays, the main market for *trans*-resveratrol is in nutraceuticals, and an increasing awareness of the possible health benefits of the chemical is likely to further increase consumer demand for the product. Consequently, it may be necessary to develop new sources for obtaining the chemical.

The various resveratrols include *trans*- or *cis*-resveratrol and the corresponding piceids. *Trans*-resveratrol is formed via the phenylalanine/polymalonate route, where stilbene synthase plays a critical step. *Trans*-resveratrol may also be transformed into *cis*-resveratrol. *Trans*- and *cis*-resveratrols may also be transformed into *trans*- and *cis*-piceids by glycosyltransferases. Glycosylation of polyphenolic compounds commonly occurs in plants to protect the plant cell from their potential toxic effects. At the same time, glycosylation may protect resveratrol from oxidation and enzymatic degradation, thus enhancing its stability [9]. Piceids are especially interesting for human health because they have also been reported to inhibit DNA synthesis in Lewis lung carcinoma cells and the formation of capillary-like tube networks (angiogenesis) of human umbilical vein endothelial cells [10]. They also inhibit thromboxane B2 in the human body [9].

Grape (*Vitis)* is one of the most important fruit crops in the world. Viticulture and enology play an important role in the economy of many developed and emerging countries. Gross world production of grape berries was estimated at more than 68.35 million metric tons in 2010 (FAO STAT Database at www.fao.org). About 27% of the grapes are consumed as fresh fruit (table grapes) and 2% as dried fruit, whereas 71% of the crop is processed, especially for winemaking.

The grapes cultivated throughout the world today mainly belong to three types, the European type (*V. vinifera* L.), the American bunch type (*V. labrusca* L.), and their derivatives, especially the hybrids obtained from *V. labrusca* and *V. vinifera*. Given the potential importance of the resveratrols, it is important to evaluate the content of resveratrols in grape germplasm in order to better utilize germplasm resources directly or in breeding programs to obtain new grape cultivars rich in resveratrols in berries and other tissues. Previous evaluations have only focused on *trans*-resveratrol in berries [11]–[14], and none have evaluated all forms of resveratrol, i.e., *trans*-resveratrol, *cis*-resveratrol, *trans*-piceid and *cis*-piceid. Although grape leaves are also rich in resveratrols [7], there are no evaluations of foliar resveratrols in grape cultivars. The objective of the present study was to evaluate resveratrol distribution in cultivars, hybrids, and species, focusing on content of all four forms in berries and leaves in order to provide information for direct use in the wine and resveratrol industries and to assist in the choice of parents in breeding programs.

## Materials and Methods

### Plant material

Seventy-five cultivars were evaluated in this study in 2009 (Table 1). These cultivars included: 34 table grapes from hybrids between *V*. *vinifera* and *V*. *labrusca*; 15 table grapes of *V*. *vinifera*; 15 wine grapes of *V*. *vinifera*; 3 interspecific juice grapes: Honey Juice (*V. labrusca* × *V. vinifera*), Beixiang (*V. thunbergii* × *V. vinifera*), Russian Concord (*V. labrusca* × *V. amurensis*) and 1 *V. labrusca* juice grape; 4 interspecific rootstock cultivars: Beta (*V. labrusca* × *V. riparia*), Shanhe 2 (*V. amurensis* × *V. riparia*), Zhi 168 (*V. monticola* × *V. riparia*) and Dog Ridge (*V. rupestris* × *V. berlandieri*), and one *V. berlandieri* rootstock cultivar, respectively. All samples were collected from the experimental vineyard of the grape germplasm repository in the Institute of Botany, the Chinese Academy of Sciences located in Beijing. All of these cultivars were planted in the spring of 1993. The grapevines were trained to cordons, spaced 1.5 m apart within the row and 2.5 m apart between rows with a north-south row orientation. They were subjected to the same management practices, such as irrigation, fertilization, soil management, pruning, and disease control.

**Table 1:** Grape cultivars used in this study.

Germplasm groups	Cultivar number	Cultivars
Table grape of LV^a^ (T-LV)	34	White Olimpia (1), Triumph (2), Dabao (3), Takasumi (4),Takatsuma (5), Guixiangyi (6), Himrod (7), Black Olimpia (8), Benni Fuji (9), Red Queen (10), Yigawa 1011(11), Beniyamabiko (12), Beniizu (13), Hanazawa Kyoho (14), Mars Seedless (15), Jiangshang (16), Venus Seedless (17), Yigawa 1055 (18), Yigawa 11015 (19), Yigawa 11060 (20), Kyoho (21), Queenora Seedless (22), Honey Red (23), Jasmine (24), Pondicherry (25), Beni Sajku (26), Fujiminori (27), Tano Red (28), Venus (29), Izunishiki (30), Zhi 180 (31), Zhiyuan 540 (32), Ziguang (33), Shigyoku (34)
Table grape of V^b^ (T-V)	15	Gros Colman (35), Misket Dounvaski (36), Flame Tokay (37), Fenniu (38), ‘Fenghuang 51’ (39), Hiro Hamburg (40), Guibao (41), Jingdajing (42), Jingfeng (43), Jingzaojing (44), Jingzhijing (45), Suffolk Seedless (46),Su 44 (47), Superior Seedless (48), Zhengzhouzaohong (49)
Wine grape of V (W-V)	15	Baiyu (50), Ugni Blanc (51), Bujiesuli (52), Meichun (53), Merlot (54), Cabernet Franc (55), Semillon (56), Suntory (57), Cabernet Gernischet (58), Lion Riesling (59), Wuyuezi (60), Chardonnay (61), Yan 73 (62), Italian Riesling (63), Zexiang (64)
Juice grape (J)	11	Honey juice (*V. labrusca* × *V. vinifera*, 65), Beixiang (*V. thunbergii* × *V. vinifera*, 66), Russian Concord (*V. labrusca* × *V. amurensis*, 67), Concord (*V. labrusca*, 68)
Wine grape of VA^c^ (W-VA)	2	Beihong (69), Beimei (70)
Rootstock grape (R)	5	Beta (*V. labrusca* × *V. riparia*, 71), Shanhe 2 (*V. amurensis* × *V. riparia*, 72), Zhi 168 (*V. monticola* × *V. riparia*, 73), Berlandier Resseguier 2 (*V. Berlandieri*,74); Dog Ridge (*V. Berlandieri*,75)

The numbers in parentheses following the cultivar indicates the accession number.

a LV, hybrids between *V*. *labrusca* and *V*. *vinifera;*

b V, V. vinifera;

c VA, hybrids between *V*. *vinifera* and *V*. *amurensis*.

Grape berries were sampled at ripening according to the former years' ripening date and as judged from seed color change to dark brown without senescence of berry tissue. Berries were sampled from three clusters, randomly chosen in three vines of each cultivar as three replicates. The leaf samples were taken from the seventh to ninth leaves of a new shoot on each grapevine with three replicates. The skins were separated by hand. All the samples of fruit skins and leaves were immediately frozen in liquid nitrogen, and then stored at −40°C.

### Extraction of resveratrols from berry skins and leaves

The frozen materials were ground to a powder in liquid nitrogen using a grinding machine (A11-b-s25, IKA, China). Each sample was extracted for 24 h in methanol and ethyl acetate (1/1, v/v) (1000 mg per 10 mL of organic solvent) at 25°C in darkness according to Liu et al. [15]. The suspension was centrifuged at 10,000× g for 10 min. The supernatant liquid was separated for collection. The remaining residue was extracted with 3 mL of methanol and ethyl acetate (1/1, v/v) and centrifuged. The two supernatants were combined and vacuum-dried with a rotary evaporator (N-1001D-WD, EYELA, Tokyo Rikakikai, Japan) at 40°C. The dried samples were then re-dissolved in 2 mL of pure methanol and stored at −40°C for resveratrol analysis.

### Measurement of resveratrols

Each liquid sample was filtered through a 0.22 µm PTFE membrane filter, then the analyses were carried out on a Dionex P680 HPLC system (Dionex Corporation, CA, USA) with a Dionex PDA-100 detector. Separation was achieved using a reverse-phase C18 column (Atlantis® T3; 4.6 mm×250 mm, 5.0 µm particle size, Waters, USA) and a guard column (Atlantis T3, 4.6 mm×20 mm, 5.0 µm cartridge, Waters, USA) maintained at 30°C with a Dionex TCC-100 thermostated column compartment, and an injection volume was 10 µL. Separation was performed at a flow rate of 1.0 mL·min^−1^ with the mobile phase consisting of H_2_O (A) and acetonitrile (B). The solvent gradient was as follows: 0–5 min from 10% to 17% solvent B; 5–12 min from 17% to 18% solvent B; 12–22 min from 18% to 22% solvent B; 22–30 min from 22% to 33% solvent B; 30–45 min from 33% to 38% solvent B; 45–50 min from 38% to 80% solvent B; 50–53 min from 80% to 10% solvent B; and, 53–60 min 10% solvent B. For fluorimetric detection, the maximum absorption wavelength of the two *trans*-isomers (*trans*-resveratrol and *trans*-piceid) was 306 nm, and the two *cis*-isomers (*cis*-resveratrol and *cis*-piceid) was 288 nm. Three replicates of each sample were also scanned from 240 to 600 nm. *Trans*-resveratrol and *trans*-piceid standards were purchased from Sigma-Aldrich (St. Louis, MO, USA) and the Chinese Standards Research Institute, respectively. The mixed solution of the *trans*-resveratrol (*trans*-res) and *trans*-piceid (*trans*-pd) standards were partly converted to the *cis*-resveratrol (*cis*-res) and *cis*-piceid (*cis*-pd) after UV-C irradiation at 6 W m^−2^ and a distance of 30 cm for 30 min. Conversion coefficients were computed from the two *trans*-isomers, respectively, and standard curves of the four isomers were made. The total resveratrol content was obtained from the sum of the four isomers.

### Graphs and data analysis

The variations of extractable amounts of the different forms of resveratrol in berry skins and leaves were analyzed, as was the distribution of the resveratrols in the samples, by S-Plus 2000 (MathSoft Inc., Cambridge, MA). Boxplots were used to display range, median, and distribution density of variables in the samples [16]. The lower and upper quartile values were indicated by the height of the box, or the interquartile range (IQR). Whiskers indicated the range of the data and were represented as vertical lines ending in a small horizontal line. Extreme values outside these whiskers were indicated by asterisks. In all the Tables and Figures, experimental data were subjected to analysis of variance using the SPSS 13.0 program (SPSS, USA.). Means were separated by Student-Newman-Keuls multiple range tests at *P*<0.05.

## Results and Discussion

### Resveratrol in grape berry skins

**Figure 1:**
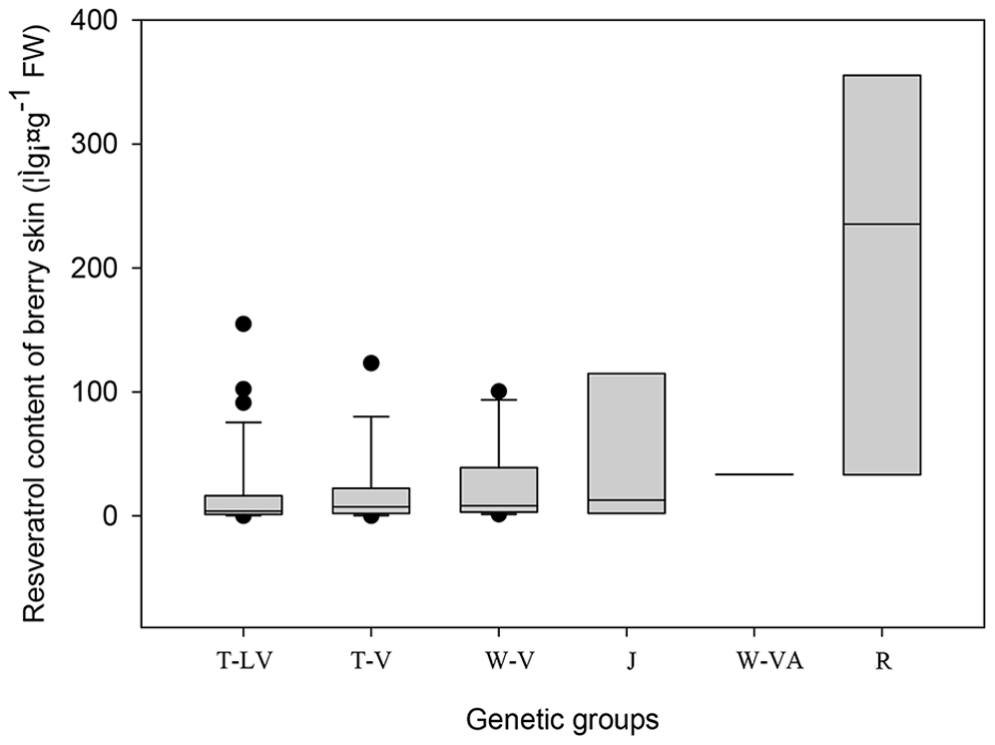
Range and distribution of total resveratrols in grape skins. The horizontal lines in the interior of the box are the median values. The height of a box is equal to the interquartile distance, indicating the distribution for 50% of the data. Approximately 99% of the data falls inside the whiskers (the lines extending from the top and bottom of the box). The data outside these whiskers are indicated by solid spots. T-LV, T-V, W-V, J, W-VA and R represent the germplasm groups as in Table 1.

The frequency distribution and median of total resveratrols in grape berry skins are shown in Fig. 1. Among all the genotypes, rootstock cultivars had the highest amounts of resveratrols in skins, ranging from 20.28 to 365.98 µg g^−1^ fresh weight (FW) with a median of 235.32 µg g^−1^ FW. However, total resveratrols in berry skins of the rootstocks varied significantly with their genetic backgrounds. The highest amounts in skin were found in ‘Berlandier Resseguier No.2’ (74) at 365.98 µg g^−1^ FW, followed by ‘Zhi168’ (73) with 344.68 µg g^−1^ FW, and ‘Shanhe 2’ (72) with 235.32 µg g^−1^ FW. Among the rootstock cultivars, ‘Dog Ridge’ (75) had the lowest amount of resveratrols with only 20.28 µg g^−1^ FW.

Juice grapes and two interspecific hybrid wine grapes from *V. vinifera* and *V. amurensis* had relatively high resveratrol content. Their medians were higher than those of table grapes. In the above juice and wine groups, ‘Concord’ (68) had very high amounts of resveratrols with 146.60 µg g^−1^ FW, while resveratrols in ‘Russian Concord’(67) were very low with only 0.5 µg g^−1^ FW.

Among most of the grape germplasm groups studied, berry skin resveratrol content was lower in the wine and table grapes of *V. vinifera*, and the interspecific hybrids between *V. labrusca* and *V. vinifera*, as compared with the previous groups. Moreover, the distribution of their totals largely skewed towards a low content with a median much lower than the average (Fig. 1). However, high resveratrol content was found in berry skins of several cultivars: ‘Venus’ (154.77 µg g^−1^ FW, 29), ‘Queenors Seedless’(102.19 µg g^−1^ FW, 22) and ‘Takasumi’(91.09 µg g^−1^ FW, 4) in hybrid table grapes between *V. labrusca* and *V. vinifera*; ‘Gros Colman’ (123.12 µg g^−1^ FW, 35) for table grapes of *V. vinifera*; ‘Cabernet Franc’(100.44 µg g^−1^ FW, 55) and Yan 73 (88.70 µg g^−1^ FW, 62) for wine grapes of *V. vinifera*.

### Resveratrols in grape leaves

**Figure 2:**
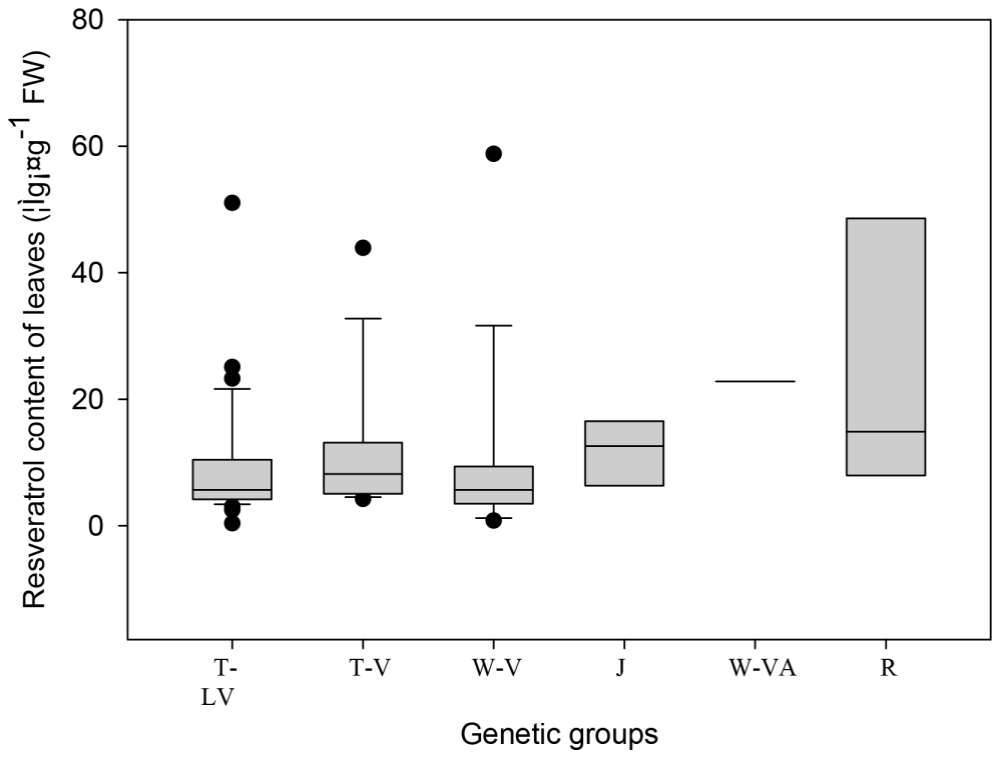
Range and distribution of total resveratrols in grape leaves. The notes about the figure and grape genotypes are the same as in Fig. 1.

The content of total resveratrols were much lower in leaves than in fruit skins (Fig. 2). Similar to fruit skins, the rootstock cultivars had the highest resveratrol content in leaves among all the grape genotypes studied, and juice and hybrid wine grapes from *V. vinifera* and *V. amurensis* had a relatively high content. All cultivars of *V. vinifera* and the hybrid cultivars of *V. labrusca* and *V. vinifera* had low total resveratrol content in the leaves.

As regards rootstocks, ‘Dog Ridge’ (75) had the highest leaf content, with 71.50 µg g^−1^ FW, and the four other cultivars had a much lower leaf resveratrol content than ‘Dog Ridge’, ranging from 10 to 30 µg g^−1^ FW. Several germplasm groups had a relatively high leaf content of total resveratrols: the hybrid wine-making cultivar ‘Beihong’ (69) with 32.63 µg g^−1^ FW, three table grape cultivars from hybrids between *V. labrusca* and *V. vinifera*, ‘Queenora Seedless’ (22), ‘Fujiminori’ (25) and ‘Ziguang’ (33) (with 51.03, 25.08 µg g^−1^ FW, and 23.26 µg g^−1^ FW, respectively), a wine grape of *V. vinifera*, ‘Zexiang’ (64) with 58.78 µg g^−1^ FW, two table grapes of *V. vinifera*, ‘Fenghuang 51’ (39) and ‘Gros Colman’ (35) (with 43.93 and 25.30 µg g^−1^ FW, respectively).

### Composition of resveratrols in berry skin and leaves

In this study, *cis*-res was not detected in any germplasm, and was thus omitted in Table 2. As shown in Table 2, the composition of berry skin resveratrols in the germplasm differed from that in leaves. In the berry skins, there are 10 cultivars which had only *trans*-res and *cis*-pd, 13 cultivars with only *trans*-res and *trans*-pd, and 5 cultivars with only *trans*-res. But, in leaves, almost all cultivars had three resveratrols, except ‘Himrod (7)’ and ‘Bujiesuli (52)’.

**Table 2:** Content (C) of different forms of resveratrols and their percentage (P) accounting for the total resveratrols in berry skins and leaves of 75 grape cultivars.

Germ-plasm group	Cultivar number	Berry skins							Leaves						
		*Trans*-res		*Trans*-pd		*Cis*-pd		Total	*Trans*-res		*Trans*-pd		*Cis*-pd		Total
		C^a^	P^b^	C	P	C	P	C	C	P	C	R	C	R	C
T-LV^c^	1^d^	0.02	100.00	-e	0.00	-	0.00	0.02	0.16	1.16	1.47	10.64	12.19	88.21	13.82
	2	1.23	25.26	-	0.00	3.64	74.74	4.87	0.05	1.66	0.55	18.21	2.42	80.13	3.02
	3	1.12	84.21	0.21	15.79	-	0.00	1.33	0.82	13.36	0.77	12.54	4.55	74.10	6.14
	4	21.99	24.14	24.49	26.89	44.60	48.96	91.09	0.05	1.06	0.37	7.86	4.29	91.08	4.71
	5	0.03	0.37	3.45	42.28	4.68	57.35	8.16	0.02	0.53	0.15	3.99	3.59	95.48	3.76
	6	6.71	77.30	0.13	1.50	1.85	21.31	8.68	0.04	1.00	0.14	3.49	3.83	95.51	4.01
	7	0.16	51.61	0.15	48.39	-	0.00	0.31	0.11	29.73	0.27	72.97	-	0.00	0.37
	8	0.33	1.70	4.45	22.93	14.63	75.37	19.41	0.36	5.15	0.20	2.86	6.43	91.99	6.99
	9	0.82	13.02	1.43	22.70	4.06	64.44	6.30	0.08	0.85	0.25	2.67	9.05	96.48	9.38
	10	0.15	100.00	-	0.00	-	0.00	0.15	0.14	2.55	0.14	2.55	5.20	94.89	5.48
	11	0.2	48.78	0.21	51.22	-	0.00	0.41	0.11	0.69	4.37	27.54	11.38	71.71	15.87
	12	3.32	26.95	0.09	0.73	8.90	72.24	12.32	0.06	0.39	0.13	0.84	15.20	98.70	15.40
	13	2.27	54.83	-	0.00	1.87	45.17	4.14	0.49	9.82	0.92	18.44	3.58	71.74	4.99
	14	0.36	11.39	-	0.00	2.80	88.61	3.16	0.08	1.59	0.10	1.99	4.84	96.41	5.02
	15	0.1	8.47	-	0.00	1.07	90.68	1.18	0.50	4.07	2.14	17.41	9.65	78.52	12.29
	16	0.79	23.44	0.94	27.89	1.64	48.66	3.37	0.07	1.75	0.20	5.00	3.73	93.25	4.00
	17	16.66	55.40	0.61	2.03	12.80	42.57	30.07	0.12	3.06	0.34	8.67	3.47	88.52	3.92
	18	1.31	51.78	-	0.00	1.21	47.83	2.53	0.05	0.68	0.31	4.20	7.01	94.99	7.38
	19	0.09	7.50	-	0.00	1.10	91.67	1.20	0.02	0.34	0.45	7.73	5.35	91.92	5.82
	20	0.35	21.34	-	0.00	1.29	78.66	1.64	0.10	1.02	0.16	1.64	9.52	97.34	9.78
	21	0.06	0.19	12.88	40.10	19.18	59.71	32.12	0.39	10.18	0.15	3.92	3.29	85.90	3.83
	22	14.55	14.24	0.39	0.38	87.25	85.38	102.1	0.18	0.35	13.38	26.22	37.48	73.45	51.03
	23	0.26	14.86	0.16	9.14	1.32	75.43	1.75	0.10	2.37	0.63	14.93	3.49	82.70	4.22
	24	0.67	87.01	0.10	12.99	-	0.00	0.77	1.05	13.32	0.47	5.96	6.35	80.58	7.88
	25	0.74	87.06	0.12	14.12	-	0.00	0.85	2.43	12.15	0.15	0.75	17.43	87.15	20.00
	26	0.37	25.17	-	0.00	1.10	74.83	1.47	2.20	36.73	0.34	5.68	3.45	57.60	5.99
	27	0.01	9.09	0.10	90.91	-	0.00	0.11	0.63	2.51	6.10	24.32	18.35	73.17	25.08
	28	0.46	10.48	0.45	10.25	3.48	79.27	4.39	-	0.00	0.28	6.62	3.95	93.38	4.23
	29	84.63	54.68	3.36	2.17	66.78	43.15	154.8	0.10	2.19	0.34	7.46	4.13	90.57	4.56
	30	0.07	0.12	14.53	24.41	44.94	75.49	59.53	0.30	6.47	0.13	2.80	4.21	90.73	4.64
	31	2.57	31.93	4.01	49.81	1.48	18.39	8.05	0.05	1.99	0.10	3.98	2.36	94.02	2.51
	32	12.91	40.12	1.10	3.42	18.17	56.46	32.18	0.02	0.21	0.09	0.93	9.58	98.86	9.69
	33	2.29	100.00	-	0.00	-	0.00	2.29	1.57	6.75	4.88	20.98	16.82	72.31	23.26
	34	7.27	48.15	0.38	2.52	7.45	49.34	15.10	0.07	1.62	0.65	15.01	3.61	83.37	4.33
	Average	5.44	38.55	3.21	15.37	14.29	46.05	18.12	0.38	5.21	1.21	10.91	7.87	83.96	9.22
T-V	35	1.03	0.84	3.56	2.89	118.53	96.27	123.1	0.02	0.08	9.82	38.81	15.46	61.11	25.30
	36	1.14	55.34	0.92	44.66	-	0.00	2.06	1.60	19.32	0.65	7.85	6.02	72.71	8.28
	37	0.89	21.71	3.21	78.29	-	0.00	4.10	1.46	20.74	1.53	21.73	4.04	57.39	7.04
	38	6.65	59.80	4.11	36.96	0.36	3.24	11.12	1.82	23.73	0.50	6.52	5.35	69.75	7.67
	39	5.12	49.66	3.14	30.46	2.06	19.98	10.31	3.24	7.38	15.87	36.13	24.83	56.52	43.93
	40	4.81	64.56	2.64	35.44	-	0.00	7.45	0.12	1.60	1.77	23.57	5.62	74.83	7.51
	41	1.16	31.35	1.33	35.95	1.20	32.43	3.70	0.08	1.58	0.78	15.42	4.20	83.00	5.06
	42	0.04	0.55	2.37	32.47	4.89	66.99	7.30	0.18	3.56	0.49	9.68	4.39	86.76	5.06
	43	4.68	16.73	12.73	45.50	10.57	37.78	27.98	0.10	0.94	3.05	28.77	7.45	70.28	10.60
	44	0.02	100.00	-	0.00	-	0.00	0.02	0.11	1.34	0.80	9.77	7.28	88.89	8.19
	45	1.25	2.44	10.79	21.07	39.18	76.49	51.22	0.58	6.21	0.79	8.46	7.97	85.33	9.34
	46	0.11	24.44	-	0.00	0.34	75.56	0.45	0.44	3.35	1.78	13.57	10.90	83.08	13.12
	47	0.18	21.43	0.66	78.57	-	0.00	0.84	0.02	0.47	0.03	0.71	4.16	98.58	4.22
	48	0.48	25.13	0.08	4.19	1.36	71.20	1.91	0.03	0.63	0.23	4.86	4.47	94.50	4.73
	49	0.62	2.78	11.64	52.27	10.01	44.95	22.27	1.02	7.25	4.41	31.34	8.64	61.41	14.07
	Average	1.88	31.78	4.40	33.25	18.85	34.99	18.26	0.72	6.55	2.83	17.15	8.05	76.28	11.61
W-V	50	0.82	27.89	0.45	15.31	1.67	56.80	2.94	0.19	12.67	0.09	6.00	1.22	81.33	1.50
	51	0.88	24.44	0.86	23.89	1.86	51.67	3.60	0.12	5.08	0.10	4.24	2.14	90.68	2.36
	52	1.81	4.30	15.45	36.73	24.80	58.96	42.06	0.06	7.59	0.74	93.67	-	0.00	0.79
	53	7.51	64.57	0.05	0.43	4.07	35.00	11.63	0.26	1.92	0.05	0.37	13.24	97.71	13.55
	54	8.44	100.00	-	0.00	-	0.00	8.44	0.12	3.46	0.07	2.02	3.28	94.52	3.47
	55	5.28	5.26	22.02	21.92	73.14	72.82	100.4	0.38	4.06	1.87	19.96	7.12	75.99	9.37
	56	0.36	33.33	0.44	40.74	0.28	25.93	1.08	0.05	0.88	0.22	3.89	5.39	95.23	5.66
	57	0.26	2.73	0.69	7.25	8.57	90.02	9.52	0.29	2.61	0.19	1.71	10.62	95.68	11.10
	58	38.69	99.59	0.16	0.41	-	0.00	38.85	0.68	8.72	0.10	1.28	7.02	90.00	7.80
	59	1.14	35.74	0.80	25.08	1.26	39.50	3.19	0.12	2.69	0.44	9.87	3.90	87.44	4.46
	60	0.36	7.39	0.34	6.98	4.17	85.63	4.87	0.15	2.55	0.33	5.60	5.41	91.85	5.89
	61	3.71	45.35	1.34	16.38	3.13	38.26	8.18	0.04	0.90	0.91	20.54	3.48	78.56	4.43
	62	25.05	28.24	8.43	9.50	55.22	62.25	88.70	0.82	10.68	0.30	3.91	6.56	85.42	7.68
	63	0.33	16.75	0.82	41.62	0.81	41.12	1.97	0.23	4.87	0.06	1.27	4.43	93.86	4.72
	64	1	75.76	0.32	24.24	-	0.00	1.32	0.83	1.41	25.34	43.11	32.60	55.46	58.78
	Average	6.38	38.09	3.73	18.03	14.92	43.86	21.79	0.29	4.67	2.05	14.50	7.60	80.91	9.44
J	65	6.43	98.92	0.06	0.92	-	0.00	6.50	0.15	3.02	0.03	0.60	4.79	96.38	4.97
	66	12.22	64.49	1.53	8.07	5.21	27.49	18.95	1.01	6.85	3.14	21.29	10.61	71.93	14.75
	67	0.01	2.00	-	0.00	0.48	96.00	0.50	0.07	0.67	0.08	0.77	10.26	98.56	10.41
	68	0.41	0.28	0.68	0.46	145.51	99.26	146.6	0.23	1.34	0.37	2.16	16.53	96.50	17.13
	Average	4.77	41.42	0.76	2.37	50.40	55.69	43.14	0.37	2.97	0.91	6.21	10.55	90.84	11.82
W-VA	69	21.17	56.85	0.21	0.56	15.86	42.59	37.24	0.54	1.65	8.34	25.56	23.74	72.76	32.63
	70	15.09	50.86	5.90	19.89	8.67	29.22	29.67	0.10	0.77	2.63	20.23	10.27	79.00	13.00
	Average	18.13	53.85	3.06	10.22	12.27	35.91	33.46	0.32	1.21	5.49	22.90	17.01	75.88	22.82
R	71	1.59	3.46	2.30	5.00	42.13	91.55	46.02	4.13	16.07	9.28	36.11	12.29	47.82	25.70
	72	7.05	3.00	12.78	5.43	215.49	91.57	235.3	0.18	5.39	0.50	14.97	2.66	79.64	3.34
	73	6.21	1.80	2.21	0.64	336.25	97.55	344.7	2.10	14.12	4.54	30.53	8.23	55.35	14.87
	74	4.04	1.10	1.82	0.50	360.12	98.40	365.9	1.07	8.53	3.23	25.74	8.25	65.74	12.55
	75	3.87	19.08	-	0.00	16.41	80.92	20.28	4.58	6.41	25.99	36.35	40.93	57.24	71.50
	Average	4.55	5.69	4.78	2.31	194.08	92.00	202.5	2.41	10.10	8.71	28.74	14.47	61.16	25.59

Notes:

a Content (C) unit is µg g^−1^FW;

b Individual resveratrol as percentage of the total resveratrol pool;

c T-LV, T-V, W-V, J, W-VA and R represent the germplasm groups as in Table 1.

d Cultivar number corresponds to the accession number in Table 1.;

e Indicates not detected.

The content of the different resveratrols varied with the genetic background. In berry skins, *trans*-res, *trans*-pd and *cis*-pd contents ranged from 0.01 to 84.63 µg g^−1^FW, from 0 to 24.49 µg g^−1^FW and from 0 to 360.12 µg g^−1^FW, respectively.

There were five cultivars whose *cis*-pd contents in berry skins were more than 100 µg g^−1^FW, especially ‘Zhi168’ (73), ‘Berlandier Resseguier No. 2’ (74) and ‘Dog Ridge’ (75) with more than 200 µg g^−1^FW. The content of *trans*-res was also high in ‘Venus’ (29) with 84.63 µg g^−1^FW. However, the content ranges of *trans*-res, *trans*-pd and *cis*-pd were 0.02–4.58 µg g^−1^FW, 0.03–25.99 µg g^−1^FW and 0–40.93 µg g^−1^FW, respectively, in leaves.

As regard the percentages of the different resveratrol components, *cis*-pd accounted for more than 90% of the total resveratrols in berry skins of rootstocks while less than 56% in the other germplasm groups (Table 2). Moreover, both *cis*-pd and *trans*-pd were not detected in ‘White Olympia’ (1), ‘Red Queen’ (10), ‘Ziguang’ (33), ‘Jingzaojing’ (44) and ‘Merlot’ (54). Interestingly, the total resveratrol content of rootstock berry skin were much higher than that in the other groups, while 5 cultivars without *trans*-pd and *cis*-pd had very low total resveratrol. Moreover, the correlation among different resveratrol types (*trans*-res, *trans*-pd and *cis*-pd) was very low in berry skins and leaves; the only high correlation coefficients (0.973) were between total resveratrols and *cis*-pd in skins (Table 3). Those results signified that the total resveratrols in berry skins were mainly from the accumulation of *cis*-pd. This was likely due to the stability of *cis*-pd in the tissues [17]. It also suggested that glycosylation, especially glycosylation of *cis*-pd, was a critical step in accumulation of total resveratrols in berry skins. In addition, *cis*-res was not detected in berry skins even though *cis*-pd was a main component, indicating that *cis*-res was rapidly glycosylated to *cis*-pd after *cis*-res was converted from *trans*-res.

**Table 3:** Correlation of resveratrols between and in grape berry skins and leaves.

	*Trans*-res	*Trans*-pd	*Cis*-pd	total
*Trans*-res	**−0.043**	0.565** y	0.473** y	0.578** y
*Trans*-pd	0.118^z^	**−0.078**	0.852** y	0.946** y
*Cis*-pd	0.141^z^	0.196^z^	**0.057**	0.973** y
total	0.293* z	0.286* z	0.983** z	**0.077**

The data on the diagonal line from top left corner to lower right corner in the table shows the correlation coefficient of the contents of individual resveratrol and total resveratrols between berry skins and leaves in all germplasms.

z Berry skin data and.

y leaf data shows the correlation coefficients among different individual resveratrols from the tissues.

* and ** indicate a significant correlation at *P*<0.05 and *P*<0.01, respectively.

The percentage of *cis*-pd was about 60% of total resveratrols in leaves of rootstock grapevines, much lower than that in berry skins (Table 2), and was even lower than that in leaves from the other groups. Moreover, almost all germplasm had all three resveratrols in leaves at low levels. There was no correlation in resveratrol content between leaves and berry skins (Table 3, diagonal). In contrast to berry skin, there were significant correlations between all three resveratrols in leaves (Table 3). From the differences in content and the proportion of each resveratrol, we suggest that the mechanism regulating the accumulation of resveratrols in leaves differs from that in berry skins. As in skins, *trans*- and c*is*-pd were also the main contributors to the total resveratrols in leaves. Gatto et al. (2008) also showed that the grape cultivars with higher resveratrol accumulate resveratrol preferentially in the glycosylated forms, *trans*- and c*is*-pd [18]. Three possible roles for resveratrol glucosides in berry skins and leaves include storage of resveratrol, transport from cytoplasm to apoplast, and protection of *trans*-resveratrol from peroxidative degradation [17].

### Principal component analysis (PCA)

**Figure 3:**
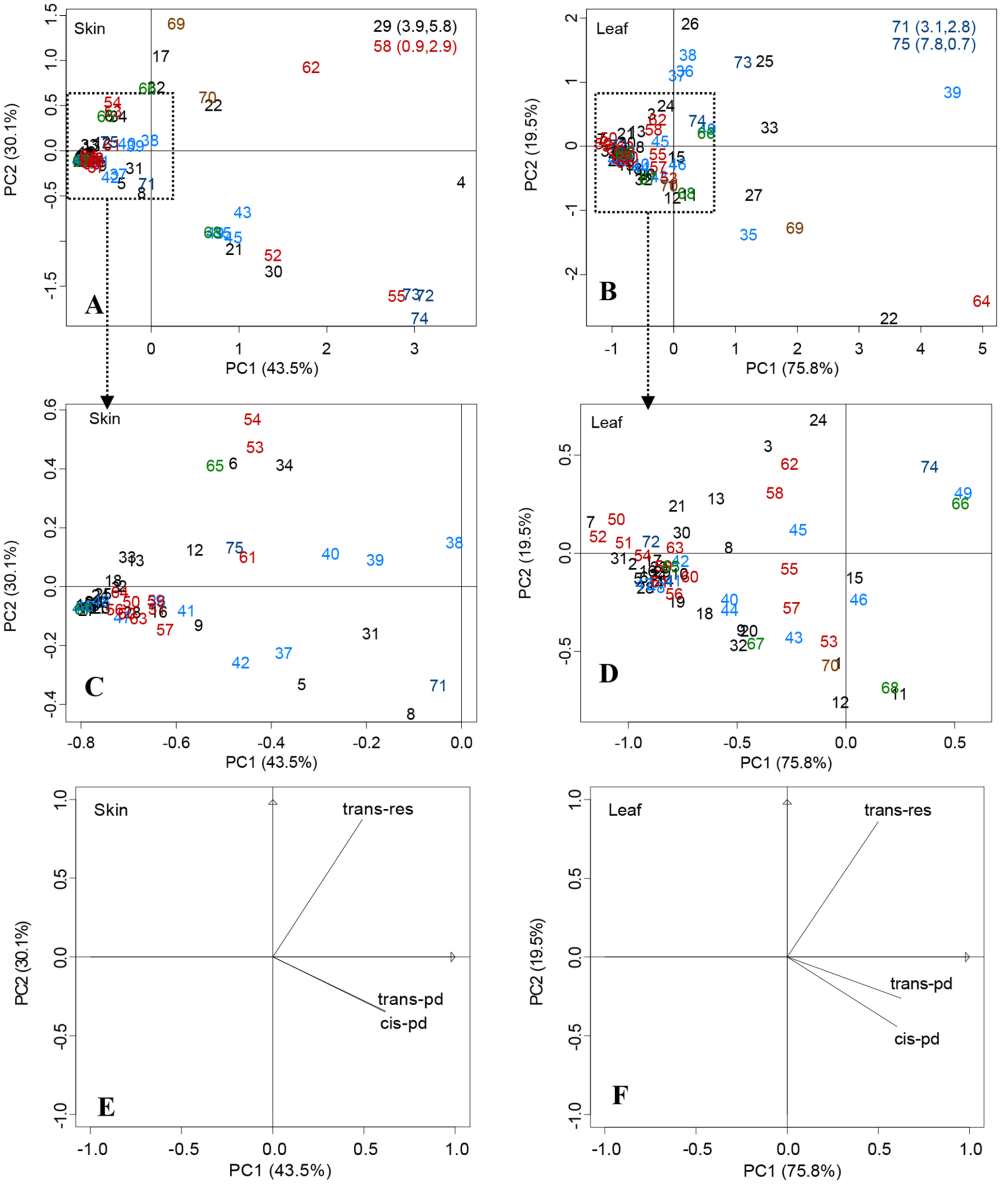
Principal Component Analysis (PCA) of seventy five grape berry skins and leaves. Fig. 3A and Fig. 3C: Scores scatter plot of PCA in berry skins; Fig. 3E: Loadings plot of PCA in berry skins; Fig. 3B and Fig. 3D: Scores scatter plot of PCA in leaves; Fig. 3F: Loadings plot of PCA in leaves. Percentages in parentheses represent the variance of each component. The numbers in the figure represent the sample numbers, which correspond to those in Table 1. Cultivars 1 to 34 are table grapes from hybrids between *V*. *labrusca* and *V. vinifera*; 35 to 49 are table grapes of *V. vinifera*; 50 to 64 are table grapes of *V. vinifera*; 65 to 68 are juice grapes; 69 and 70 are wine grapes of *V. vinifera* × *V. amurensis*; and, 71 to 75 are rootstock cultivars.

PCA was used to analyze the data for *trans*-res, *trans*-pd and *cis*-pd from berry skins and leaves of 75 grape cultivars. Fig. 3 shows the relationships between the cultivars (scores) and their resveratrols (loadings). The two PCs carried a large amount of important information and accounted for 73.6% and 95.3% of the total variance for resveratrols in berry skins and in leaves, respectively. The PCA scatter plots of berry skins and leaves are shown in Fig. 3A and Fig. 3B, respectively, and the corresponding loading plots, which establish the relative importance of the variables, are shown in Fig. 3E and Fig. 3F, respectively.

With regard to resveratrols in berry skin, the grape cultivars can be divided into four groups based on the positions in the PCA scatter plots (Fig. 3A). Group 1: The group situated in the low right part of the third quadrant was comprised of three rootstock cultivars, ‘Shanhe 2’ (72), ‘Zhi168’ (73), ‘Berlandier Resseguier No.2’ (74); and one wine-making cultivar ‘Cabernet Franc’ of *V. vinifera* (55). This group was characterized by a very high content of *cis*-pd. Group 2: The group was also situated in the left part of the third quadrant and was comprised of three table grape cultivars from hybrids between *V. labrusca* and *V. vinifera* [‘Takasumi’ (4); ‘Kyoho’(21); ‘Izunishiki’(30)],four table grape cultivars of *V. vinifera* [‘Gros Colman’(35); ‘Jingfeng’(43); ‘Jingzhijing’(45) and ‘Zhengzhouzaohong’(49)], one wine-making cultivar of *V. vinifera* [‘Bujiesuli’ (52)], and one juice cultivar from *V. labrusca* [‘Concord’ (68)]. This group was characterized by a relatively higher content of *trans*-pd or *cis*-pd. Group 3: This group was situated mainly in the fourth quadrant and consisted of three table grape cultivars from hybrids between *V. labrusca* and *V. vinifera* [‘Venus Seedless’(17); ‘Queenora Seedless’(22); ‘Venus’(29)], two wine-making cultivars of *V. vinifera* [‘Cabernet Gernischet’(58); ‘Yan 73’,(62)], and two wine-making cultivars from hybrids between *V. vinifera* and *V. amurensis* [‘Beihong’(69); ‘Beimei’(70)]. This group was characterized by a very high content of *trans*-res. ‘Venus’ (29), which is a hybrid between *V. vinifera* and *V. labrusca*, was in the top right quadrant which indicates that it is associated with an extremely high positive value for PC1 and PC2. This cultivar was characterized by an extremely high *trans*-res. Group 4: The other *Vitis* cultivars were found near the PC1 axis. Their *trans*-res, *trans*-pd and *trans*-pd contents were very low.

As shown in Fig. 3B for leaves, the grape cultivars can be divided into four groups based on positions in the scores scatter plot of PCA. Group 1: This group was situated mainly in middle and lower right part of the third quadrant and consisted of two table grape cultivars from hybrids between *V. labrusca* and *V. vinifera* [‘Queenora Seedless’(22); ‘Fujiminori’(27)], one table grape cultivar of *V. vinifera* [‘Gros Colman’ (35)], one wine-making cultivar of *V. vinifera* [‘Zexiang’ (64)], and one wine-making cultivar from a hybrid between *V. vinifera* and *V. amurensis* [‘Beihong’ (69)]. This group was characterized by higher contents of *trans*-pd and *cis*-pd. Group 2: This group was situated primarily in the left and right parts of the four quadrants and was comprised of three table grape cultivars from hybrids between *V. labrusca* and *V. vinifera* [‘Pondicherry’(25); ‘Beni Sajku’(26); ‘Ziguang’(33)], four table grape cultivars of *V. vinifera* [(‘Misket Dounvaski’(36); ‘Flame Tokay’(37); ‘Fenniu’(38); ‘Fenghuang 51’(39)], and three rootstock cultivars [‘Beta’(71); ‘Zhi168’(73)]. This group was characterized by a higher *trans*-res content. Group 3: This group was situated in the right side of the fourth quadrant and was comprised of the rootstock grape *V. Berlandieri ‘*Dog Ridge’(75) with higher *trans*-res, *trans*-pd and *cis*-pd. Group 4: The other *Vitis* cultivars were found near the PC1 axis. Their *trans*-res, *trans*-pd and *cis*-pd contents were very low.

## Conclusions

Resveratrol content varied significantly with genetic background as well as their usage. *Trans*-res, *cis*-pd and *trans*-pd were detected in berry skin and leaves, but *cis*-res was not. Generally, resveratrol totals were lower in leaves than berry skins in most cultivars. Rootstock cultivars had a higher content of total resveratrols than the other cultivars. *Cis*-piceid was the most abundant resveratrol in most cultivars, while *trans*-res and *trans*-pd were minor components. Some cultivars were found to have extremely high or low *trans*-res, *cis*-pd, *trans*-pd or total resveratrols in berry skins or leaves. There were no significant correlations of total resveratrols between berry skins and leaves.
